# Application of the Supportive Accountability Model in Digital Health Interventions: Scoping Review

**DOI:** 10.2196/72639

**Published:** 2025-09-26

**Authors:** Gary Kwok, Shannon Pui Ying Cheung, Jennifer Duffecy, Katie A Devine

**Affiliations:** 1 Cancer Prevention Precision Control Institute Center for Discovery and Innovation Nutley, NJ United States; 2 Department of Psychiatry College of Medicine University of Illinois - Chicago Chicago, IL United States; 3 Rutgers Cancer Institute of New Jersey New Brunswick, NJ United States

**Keywords:** supportive accountability, human support, digital health intervention, mHealth, intervention engagement, intervention adherence

## Abstract

**Background:**

Digital health interventions (DHIs) harness technological innovation to address challenges in the accessibility and scalability of health care. However, the effectiveness of DHIs is challenged by low user engagement and adherence, as users tend to drop out over time. The supportive accountability model (SAM) is a theoretical framework designed to enhance adherence to DHIs by incorporating structured human support.

**Objective:**

Guided by SAM, this scoping review answers the following research questions: (1) What is the extent of research on human support factors and their influence on engagement with and adherence to DHIs? and (2) What is the extent of research applying SAM (ie, accountability, bond, and legitimacy) to improve engagement with and adherence to DHIs?

**Methods:**

Our search strategy followed the PRISMA-ScR (Preferred Reporting Items for Systematic Reviews and Meta-Analyses Extension for Scoping Reviews). We conducted our literature search using 6 databases selected based on relevance to our research topic: MEDLINE, PsycINFO, Embase, CINAHL, Scopus, and ClinicalTrials.gov. Search terms included (“human support” OR “supportive accountability”) AND (engagement OR adherence) AND intervention, applied to titles, abstracts, and keywords. Hand-searching was also used to identify additional relevant articles. Two authors (SPYC and GK) screened articles in multiple rounds using predefined inclusion and exclusion criteria. The final sample consisted of 36 empirical, peer-reviewed articles published in scholarly journals. All articles examined human-supported DHIs.

**Results:**

Implementation of human support among the interventions varied by the source, delivery method, and frequency and duration of support. Overall, there were inconsistencies in the application of SAM to intervention designs. Support was provided by 4 main groups: peers and peer specialists, health experts and practitioners, trained coaches, and members of the research study team. Modes of communication included phone or video calls, as well as text-based support, such as messaging or email. The frequency and duration of support varied across studies and were influenced by the communication method used, with more structured and frequent contact occurring in interventions that relied on synchronous support, such as phone or video calls. In addition, we found that some studies used human support as the primary mode of intervention delivery rather than as an adjunctive tool, focusing on improving engagement and adherence, as proposed by SAM. Aside from accountability, there was also a lack of explicit focus on other constructs within the model (eg, bond and legitimacy).

**Conclusions:**

This scoping review highlights the current use of human support to promote DHI adherence and reveals gaps in the application of SAM. Future research should address all core SAM components—not just accountability—and ensure human support is used as an adjunct to enhance engagement. These steps can help maximize the impact of DHIs on health care access and outcomes.

## Introduction

### Digital Health Interventions

Digital health interventions (DHIs) encompass a range of technologies and applications designed to enhance health outcomes, improve health care delivery, and promote patient engagement. DHIs leverage advancements in mobile health (mHealth), telemedicine, wearable devices, and health information systems to transform the health care landscape by offering scalable, accessible, and cost-effective solutions for health concerns such as cardiovascular disease and mental illness [1-4]. However, the application of DHIs is challenged by low user engagement and adherence, or the “law of attrition,” a phenomenon in which users tend to drop out of eHealth interventions over time [5]. User engagement refers to the degree to which users interact with the intervention [6], while adherence reflects the extent to which users follow prescribed usage patterns [7]. Because both are crucial factors in determining the effectiveness of DHIs [8,9], it is imperative to understand the factors related to engagement and adherence.

### Theoretical Foundations: Self-Determination and Social Cognitive Theories

The influence of DHIs on behavioral change, including user engagement and adherence, is grounded in several psychological and behavioral theories. Two such theories—self-determination theory (SDT) and social cognitive theory (SCT)—offer complementary insights into motivation, self-regulation, and social influence in the context of DHIs.

SDT posits that social environments and interventions are most effective when they support 3 basic psychological needs: autonomy (the sense that one’s behavior is self-directed and volitional), relatedness (feeling connected to others), and competence (feeling effective and capable of achieving goals) [10]. When these needs are met, individuals are more likely to internalize motivation, resulting in lasting behavioral change. SDT views motivation as a continuum ranging from amotivation (no motivation) to intrinsic motivation (engagement driven by inherent interest or enjoyment) [10]. In between lie controlled regulation (including external and introjected regulation) and autonomous regulation (including identified, integrated, and intrinsic regulation). Behaviors driven by more autonomous forms of motivation are generally more persistent and robust across time [11].

SCT similarly focuses on the internal and social processes that guide behavior, emphasizing self-regulation as a key mechanism. According to SCT, behavior is shaped through a dynamic interplay between personal factors (such as beliefs and self-efficacy), environmental influences (including social norms and reinforcement), and behavioral capabilities (acquired through observation and experience) [12,13]. Central to SCT is the construct of self-efficacy—one’s belief in their ability to perform a specific behavior—which influences the goals people set, the effort they expend, and their persistence in the face of challenges. SCT also emphasizes the role of observational learning, outcome expectations, and social support in shaping health behaviors, making it especially relevant to DHIs that incorporate human support or social features [14].

### Human Support Factors in DHIs

Human support has been identified as an important factor for enhancing both engagement with and adherence to DHIs. Human support factors encompass various forms of support, including social support from peers, human coaching and tailored support, and the involvement of health care providers. These collectively create an environment that motivates users to engage with and adhere to interventions [15,16]. By providing users with accountability and feedback, human support can significantly improve the outcomes of DHIs [17,18]. For example, several studies have highlighted the importance of peer support for enhancing engagement with DHIs. Peer relationships uniquely create reciprocal accountability, wherein users hold each other responsible for achieving goals (eg, see [19]). Peer support can foster autonomy, shared experiences, and a sense of belonging, collectively influencing engagement through mechanisms such as goal setting, task agreement, and social bonding [19]. In particular, peer support offers a unique type of support; for example, peer support specialists may include certified peer specialists or recovery coaches who share a similar mental health diagnosis, are in recovery, and provide peer support services [20]. They can leverage familiarity, perceived similarity, and trust [21] to develop a bond with consumers [22] that facilitates engagement with DHIs [23].

The application of human coaching in DHIs has also demonstrated a positive impact on user engagement. Coaches can provide personalized guidance, address challenges, and motivate users to continue using the intervention [24]. Tailoring coaching to individual needs and preferences can amplify its impact. Similarly, health care providers can play a significant role in influencing user engagement with DHIs. Providers can introduce and promote DHI use to their patients, offer guidance on integrating DHIs into existing treatment plans, and address any concerns or questions that may arise [25]. Ongoing support from a trusted health care professional can increase user buy-in and commitment to the intervention [26].

Human support can play a crucial role in fulfilling the basic psychological needs outlined in SDT, thereby enhancing engagement with DHIs and promoting lasting behavior change. By fostering autonomy, competence, and relatedness, supportive interactions can enhance individuals’ intrinsic motivation, which is linked to higher levels of adherence. For instance, personalized feedback and encouragement from health care providers can strengthen users’ sense of competence in managing their health. In a study by Höchsmann and colleagues [27], an mHealth intervention designed for individuals with type 2 diabetes incorporated autonomy-supportive features—such as user-driven goal setting—and tailored recommendations to meet individual needs. Their findings demonstrated improved adherence in the intervention group compared with the control group [27].

Similarly, support from peers and health care professionals can provide powerful modeling and reinforcement, key mechanisms in SCT that help sustain user engagement and adherence [28]. A review of SCT-based interventions found that incorporating social support features, such as peer support or coaches, was associated with improved health outcomes [29]. Emerging research also highlights the synergistic potential of combining SDT and SCT principles in the design of DHIs. For example, Smith and colleagues [30] developed a mobile app for obesity prevention among adolescent boys in low-income communities that integrated goal-setting tools (supporting SDT principles of autonomy, competence, and relatedness) with social comparison features (reflecting SCT’s concept of observational learning). Compared with the control group, participants using the app showed significant improvements in muscular fitness, movement skills, and key weight-related behaviors [30].

### Supportive Accountability Model

One theoretical framework that has gained prominence in understanding the role of human support in DHIs is the supportive accountability model (SAM) [31]. SAM suggests that providing accountability in a supportive manner can greatly enhance user engagement and adherence [31]. SAM suggests that having human support who can provide encouragement, monitor progress, and hold users accountable can boost the intrinsic motivation of users, resulting in increased sustained engagement and adherence to the intervention. SAM proposes 3 interpersonal or human supportive factors that could lead to better adherence: accountability, bond, and legitimacy.

*Accountability* refers to the expectation that an individual may have to justify their actions to their accountability partners [32]. In SAM, accountability is established through reinforcing social presence, expectations (ie, goal setting), and performance monitoring. Accountability and DHI adherence can then be enhanced by the *bond* between the human support and intervention participant, as well as *legitimacy* (ie, trustworthiness, benevolence, and expertise) [31]. In short, people adhere better when they are held accountable, particularly when they are held accountable by people they respect and care about [33]. Drawing from SDT, the concept of autonomous accountability —an internal, self-endorsed motivation to meet expectations—has been posited to yield more sustainable adherence than controlled accountability, which is driven by external pressure or obligation [34]. Oussedik et al [34] furthered developed autonomous and controlled accountability in a modified version of Bandura’s [12] SCT, illustrating how social norms, interpersonal interactions, and supportive relationships influence accountability and, in turn, behavioral adherence. Accountability can also be conceptualized through self-observation, a core SCT process wherein individuals monitor their behaviors with the awareness that others are observing their progress. Through this lens, accountability may foster a greater sense of personal responsibility and satisfaction as individuals self-regulate their behaviors to achieve their health goals [12].

Studies applying SAM in DHIs have demonstrated its effectiveness in various contexts. For instance, mental health DHIs that incorporate supportive accountability from therapists or coaches have shown higher rates of user adherence and better clinical outcomes [35,36]. Similarly, DHIs for weight management that include regular check-ins with a coach have resulted in more significant weight loss and improved adherence than those without such support [37,38].

### Taxonomy of DHIs

With the rapid advancements in the field of DHIs, there is a need to categorize DHIs based on their core components, such as the type of support provided, level of personalization, and mode of delivery, to guide the conceptualization and development of future DHIs. Pineda et al [39] developed a framework to present 4 types of digital mental health interventions (DMHIs) by categorizing interventions based on the level of human involvement, from primary content delivery (ie, high involvement) to adjunctive support (ie, low involvement). The taxonomy is categorized into the following types: (1) provider-administered DMHIs (asynchronous or synchronous delivered by practitioners via a digital platform); (2) provider-administered DMHIs with blended digital adjuncts (asynchronous or synchronous delivered by practitioners via a digital platform with digital adjuncts to enhance the interventions); (3) self-help DMHIs with human support (offering human support to self-help digital tools to reduce the substantial proportion of dropouts); and (4) self-help fully automated DMHIs (without human support).

The taxonomy highlights that human-supported interventions, particularly those incorporating elements of SAM, can achieve better engagement and adherence outcomes in self-guided or automated interventions, or what the authors term “Type 3: Human-supported self-help interventions” [39]. This clear distinction is important as it guides how SAM is applied in research. Again, this highlights the crucial role of human support in the effective implementation of DHIs, offering a structured approach for evaluating and designing these interventions.

Despite the recognized importance of human support and the promising potential of SAM, the extent to which these factors have been applied in DHI studies remains unclear. The goal of this scoping review was to answer the following questions: (1) What is the extent of research on human support factors and their influence on engagement with and adherence to DHIs? (2) What is the extent of research applying SAM (ie, accountability, bond, and legitimacy) to improve engagement with and adherence to DHIs? Results can guide future research and inform the design and implementation of DHIs that encourage adherence, thereby optimizing the efficacy of interventions in promoting health and well-being.

## Methods

The study procedure was informed by the scoping review methodological framework described in Arksey and O'Malley [40]. This framework follows a 5-stage approach: (1) identifying guiding research questions, (2) searching for relevant studies, (3) selecting those to be included, (4) charting data, and (5) collating and summarizing data and reporting results. This review protocol was not registered prior to beginning the review.

### Stage 1: Identifying Research Questions

Using SAM [31] as a guiding framework, we developed the following research questions to investigate: (1) What is the extent of research on human support factors and their influence on engagement with and adherence to DHIs? and (2) What is the extent of research applying SAM (ie, accountability, bond, and legitimacy) to improve engagement with and adherence to DHIs?

### Stage 2: Identifying Relevant Studies

Our search strategy followed the PRISMA-ScR (Preferred Reporting Items for Systematic Reviews and Meta-Analyses Extension for Scoping Reviews) framework [41]. First, the following 6 databases were selected for our literature search: MEDLINE, PsycINFO, Embase, CINAHL, Scopus, and ClinicalTrials.gov. These databases were selected based on their relevance to the topic.

The following search terms were entered into each database’s search engine in February 2024: (“human support” or “supportive accountability”) and (engagement or adherence) and intervention. The terms “human support” and “supportive accountability” were grounded in the SAM [31] and reflect core constructs relevant to human-supported DHIs. “Adherence” was informed by the MeSH term “adherence interventions,” which encompasses strategies aimed at improving compliance with health interventions. “Engagement” is a controlled vocabulary term commonly used in digital health literature to describe user interaction with interventions, including in journals such as the Journal of Medical Internet Research (eg, “user engagement”). These terms were searched within article titles, abstracts, and keywords. When the options were available, search results were limited to articles from peer-reviewed journals, empirical studies, and articles written in English. We also excluded reviews (ie, scoping, systematic, narrative) when possible since one of our inclusion criteria (listed in the next section) was that the article must be an empirical study. The results of each search were exported into database-specific Excel (Microsoft Corp) sheets that contained article metadata, including title, year, author(s), journal, and abstract.

Additionally, we conducted a hand search by reviewing the “Cited By” and references lists of included articles to identify additional relevant studies. This manual step helped address limitations in database indexing and variations in author-assigned keywords that may have excluded relevant articles from our initial search. Data extraction was conducted by the second author (SPYC). The independent review process for study selection, including how discrepancies were resolved between raters, is detailed in the next section.

### Stage 3: Selection of Studies

**Figure 1:**
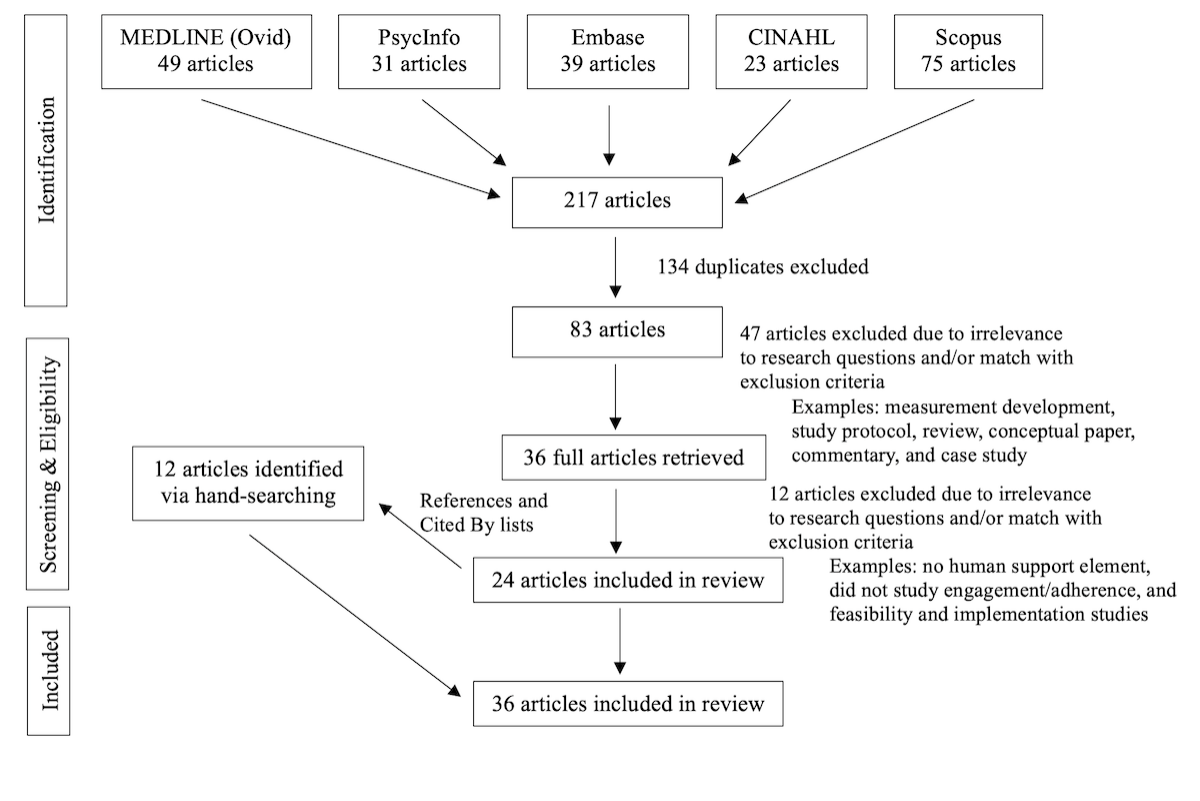
Flow diagram of the search strategy.

Our search yielded 217 articles across all 6 databases. The number of results generated in each database search is shown in Figure 1. Our search using ClinicalTrials.gov yielded 0 results and was therefore excluded. Among the 217 articles, 134 duplicates were removed, resulting in 83 remaining articles.

The study team (GK and SPYC) reviewed all the article abstracts to screen for relevance and eligibility. Inclusion criteria included that the article was (1) peer-reviewed, (2) published in a scholarly journal, (3) about an empirical study (quantitative, qualitative, or mixed methods), (4) a study that focused on DHIs that used human support, (5) written in English, and (6) full text available online. Criteria (1) and (2) ensured that the included articles had undergone peer review and met a basic standard of a systematic process and scientific rigor, which resulted in excluding white papers, theses, and dissertations. Criterion (3) excluded nonempirical works such as reviews, commentaries, study protocols, conceptual or theoretical papers, and papers on intervention development because our guiding questions focus on the application of a theoretical framework in empirical research. Criterion (4) was required to ensure relevance to our guiding questions about research using SAM, a theoretical framework that is centered around the role of human support in DHI adherence. Finally, criteria (5) and (6) were decided upon for pragmatic reasons, as the authors needed to be able to access and download the full articles as well as to read them.

The 83 abstracts were independently reviewed by the first two authors (SPYC and GK) based on the inclusion criteria and relevance to our research questions. Following the initial screening, they met to discuss which articles should be excluded. In cases of uncertainty or disagreement, the full text was retrieved and consulted to determine whether the study adequately addressed the guiding research questions [42]. Only after both authors reached a consensus on the article was it included or excluded at this stage. Among the 83 articles, 47 were excluded due to irrelevance, and 36 full articles were downloaded for further review. Among these excluded articles were measurement validation studies, study protocols, scoping and systematic reviews (not an empirical study), conceptual papers, commentaries, and a case study. These were excluded either because they were not an empirical study or because they did not study engagement with or adherence to a DHI.

The first two authors independently reviewed each full article, noting those that were unrelated to the research questions. In another peer debriefing, the team met to determine the final sample. We excluded 12 additional articles due to irrelevance, including interventions that did not use a human support element, did not examine engagement or adherence, or focused mainly on feasibility and implementation outcomes. These were considered irrelevant because they did not reflect key concepts of SAM. To supplement the sample, the team reviewed the reference lists and Google Scholar “Cited by” lists of the remaining 24 articles. Reference lists had an average of 45.8 citations (median=40), while Cited By lists, retrieved via Google Scholar in February 2024, had an average of 51.9 articles (median=34.5). We identified 12 more articles through this hand-searching strategy, resulting in a final analytic sample of 36 articles.

### Stage 4: Charting the Data

The narrative review tradition typically applies an analytical framework to standardize data collection [40]. In addition to collecting conventional metadata (eg, title, author, year, journal, findings), information about the key components of SAM (ie, accountability, legitimacy, and bond) was also collected. The following data were collected from each article: title, author(s), year, journal, study design, stage of study (clinical trials), measurement instruments used (if any), engagement or adherence measure (if any), sampling strategy, sample size, study location (country), study population (age and health status), engagement model (if any), intervention target outcome, human support strategy, presence of a training description, findings related to engagement and adherence, source of human support, frequency of human support interactions, mode of communication, duration of interaction, and comparison group(s).

### Stage 5: Collating, Summarizing, and Reporting Results

The second author (SPYC) first identified preliminary themes from the data charted in Stage 4. After preliminary themes were generated, the first author (GK) and second author (SPYC) continued to develop and refine the themes until both authors agreed that they captured the review’s findings. Strategies for rigor include team debriefings and an audit trail to keep track of decisions made throughout the analysis [43].

## Results

### Description of Studies

The results of the search are shown in Figure 1. The distribution of research designs among the articles included in our scoping review (N=36) is as follows: 25 (69%) were quantitative, 7 (19%) were mixed methods, and 4 (11%) were qualitative studies. Most studies (30/36, 83%) were conducted with adult participants, while only 6 (6/36, 17%) examined engagement in interventions with youth. Two-thirds (24/36, 67%) of the studies were conducted in the United States. Other study locations included Australia and New Zealand, the Netherlands, Spain, Norway, Malaysia, Singapore, England, and the United Kingdom. Over one-third (14/36, 39%) of the studies were randomized controlled trials, while an additional 2 studies were randomized trials.

### Human Support: Sources, Mode of Communication, Frequency, and Duration

#### Overview

The implementation of human support among the interventions varied by the source of the support (ie, who is providing the support?), the delivery of the support (ie, how do human supports contact the participants?), and the frequency and duration of the support (ie, how often and how long is each contact?). We found that sources of human support across interventions could be categorized into 4 groups: (1) peers and peer specialists; (2) health experts and practitioners; (3) platform or product experts and trained coaches; and (4) members of the research study team. The frequency and duration of contact between human support and the participants (eg, weekly, 3 times a week) appeared to be related to the mode of communication (eg, phone calls, text messaging). For example, text messages occurred more frequently than phone calls.

#### Sources

##### Peers and Peer Specialists

Studies by Blonigen et al [44], Duffecy et al [45], Duffecy et al [46], Duffecy et al [47], Ho et al [48], Lattie et al [49], Lederman et al [50], Possemato et al [51], and Tomasino et al [52] used peer support to engage participants in their respective DHI studies. Of these, 7 [45-50,52] integrated social networking features into their intervention platforms, which are comparable to those found on popular social media sites such as Facebook. For example, these features allowed participants to view each other’s activity on the platform [45,47-50,52], create and reply to each other’s posts [47,49,50,52], and send notifications, sometimes referred to as “nudges,” to peers who had been inactive recently [45,48,49,52]. The notification feature would remind inactive users to reengage with intervention tasks, such as viewing educational content or completing exercises on the platform [45,48,49,52].

In studies wherein human support was delivered via social networking features, support came from peers who were also participants in the study. Meanwhile, Blonigen et al [44] used peer specialists who were not participants in the studies to increase engagement. The peer specialists were employed by the Veterans Health Administration to provide human support to participants. In the Veterans Health Administration, peer specialists are veterans who are in recovery from substance use or mental health problems and are trained to provide services to other veterans who are struggling with similar problems using their lived experience [53]. Their role includes providing patients with emotional support, serving as role models for self-management of health issues, and assisting patients with navigating the health care system. In this study, peer specialists would use their lived experience and support to facilitate participants’ engagement with the intervention for unhealthy alcohol use. This type of peer support emphasizes mentorship from those who have more experience overcoming a challenge, contrasting with the former interventions, which relied on cohort members to support one another in engaging with the interventions.

##### Health Experts and Practitioners

Of the 36 studies, 10 (27.7%) investigated interventions wherein various health experts and practitioners provided human support. Health professionals included nurses and nurse interns [54,55]; a behavioral weight loss interventionist [56]; dietitians [57]; mental health providers, such as clinical psychologists [57,58]; therapists [59-61]; and clinic staff [49,50]. Interventions used by Chhabria et al [57] and Jesuthasan et al [24] also drew human support from experts from multiple disciplines. For example, Jesuthasan et al [24] used a multidisciplinary team comprising dietitians, physiotherapists, fitness coaches, pharmacists, and medical advisors to deliver various components of the intervention, providing individualized therapeutic content to participants.

##### Trained Coaches

Although the term “coach” may broadly encompass various disciplines (eg, peer specialists, health experts and practitioners, and platform or product experts), coaches (eg, a recovery coach) do not necessarily have professional training related to the content of the intervention. Therefore, coaches often receive additional training tailored to the intervention. Of the 36 studies, 14 (39%) studies explicitly used the term “coach” to describe the sources of human support in their studies [15,38,58,62-71]. One additional study described their human support as “telephone counselors” [72], who were trained by the study team to provide support and advice, facilitate problem-solving, and promote participants’ use of the intervention platform. Similarly, in a commercialized intervention platform (Naluri), Jesuthasan et al [24] employed and trained the delegated staff to provide support for their users.

##### Research Study Team

Like coaches, research team members were not professionally trained for the content of the intervention. However, their roles as human support were less focused on the intervention content and more focused on research study-specific questions or tasks. In 3 (3/36, 8%) studies, members of the research team provided general support to participants [73-75]. In the study by Cheng et al [73], participants were guided through the intervention by an animated digital therapist with interactive components; research staff, however, were available to participants via phone or email for inquiries about research-related processes, such as creating an account on the intervention platform. In the study by Glasgow et al [74], study team members provided initial support to participants during the early phase, while later support came from a diabetes care manager or a nutritionist. In the study by Kelders et al [75], the research team members wrote automated SMS text messages and scheduled them to be sent to participants 3 times a week.

#### Mode of Communication, Frequency, and Duration

The frequency and duration of contact with those providing human support varied and were often determined by the mode of communication (Multimedia Appendix 1). Several interventions also offered participants options from which to select their preferred method of communication with human support.

##### Phone and Video Chat

Among the 23 (23/36, 64%) interventions in which human support contacted participants via phone call or video chat, 10 (10/23, 44%) studies involved weekly contact, and 2 (2/23, 9%) involved contact every other week. One intervention (1/23, 4%) involved a tapered frequency, beginning with weekly contact for 8 weeks, then gradually decreasing to monthly for 2 months [56]. Four interventions (4/23, 17%) scheduled contact based on the intervention curriculum and schedule. For example, human support in the study by Dennison et al [38] contacted participants after weeks 1 and 4, while human support in the study by Glasgow et al [74] contacted participants after weeks 2 and 8, as well as at a 4-month follow-up. Coaches in the study by Baron et al [76] contacted participants every 2 modules that the latter completed. In another study, participants received 5 peer support sessions throughout the 8-week intervention [51]. Two interventions that used only phone-based support offered more frequent contact, ranging from twice a week [59] to 5 times a week [65]. The duration of interactions between human support and participants was reported by 15 (15/23, 65%) studies. Across these studies, contact lasted between 5 minutes and 50 minutes. Of these 15 studies, 3 (20%) studies began with longer sessions to build rapport between the support and participants, as well as to orient participants to the intervention. These initial “engagement” sessions were about 20 minutes in the study by Baron et al [76] and 30 minutes to 45 minutes in the studies by Mohr et al [70,71]. Of the 15 studies, 2 (13%) studies provided support through group-based videoconferencing sessions, which lasted approximately 20 minutes to 30 minutes [15,64].

##### Text-Based Support

In total, 23 (23/36, 64%) interventions used or offered text-based human support through SMS messaging, emails, private messaging, or forum posts on the intervention platform. Text-based communication, which tends to be asynchronous, allowed for a greater frequency of participant interactions with human support. In fact, 13 of the 23 (57%) text-based support interventions used an asynchronous model of continuous support, enabling participants to send messages to their human support at any time. On the other hand, 3 studies [15,64,65] used synchronous text messaging exchanges. For example, Sayegh et al [65] reported 3 to 5 text conversations a week, each lasting approximately 5 minutes. Some studies (4/23, 17%) used text-based communication as a supplementary form of support between calls, enabling participants and coaches to remain connected in the interim [52,68,70,71].

#### Applying SAM

##### Use of SAM

Although we devised our inclusion criteria for this scoping review based on SAM by Mohr et al [31], not all studies indicated SAM as a guiding model for their intervention designs. Of the 36 studies, 22 (61%) studies justified the incorporation of a human support component using SAM (see Multimedia Appendix 2). Although 4 (4/36, 11%) others did not explicitly identify SAM, the seminal article by Mohr et al [31] was cited [49,52,58,69]. Mohr was a co-author in 2 of these studies [49,52]. The following section focuses on the extent to which accountability, legitimacy, and bond were incorporated in the 22 studies that specifically identified SAM when describing intervention development and considerations for enhancing engagement.

##### Accountability

Nearly all studies but one (21/22, 96%) described how the intervention design encouraged accountability. The most common strategies included checking in (social presence), discussing intervention usage (expectation—process accountability; eg, [15,44,56,64,70,71]), reviewing and setting personal goals (expectation—goal setting; eg, [36,54,59,62,70]), and creating plans for continued engagement and progress toward goals (goal setting; eg, [63,72,74]). In some cases, these strategies were coupled with monitoring of participants’ activities on the intervention platforms (performance monitoring), such as mobile apps or websites (eg, [46,47,50,55,63,72,76]). Among the interventions that relied on mutual peer support from participants, the use of social media features such as news feeds allowed participants to be accountable to one another (social presence). For example, participants could view when their peers were completing activities on the platform [24,46-48,50,76]. On some platforms, participants had the ability to send push notifications to peers who had been inactive, prompting them to engage with the intervention [46-48]. In 1 study [73], accountability was reinforced by quizzes (expectation—outcome accountability*)* and email notifications (social presence). Kelders et al [75] highlighted that human support could provide opportunities for interactions by asking questions and sending personalized responses.

##### Legitimacy

People are more likely to respond positively to accountability demands from a coach who is perceived as legitimate [77]. Legitimacy can be categorized into instrumental (ie, expertise and reciprocity) and relational factors (ie, trustworthiness and benevolence) [77,78]. Of the 4 characteristics, only expertise was considered most among the articles accounted for in this review. Expertise was derived from training or other credentials, such as profession/discipline or license/certification (eg, [24,54,58,60-63,66,69-71]). On the other hand, studies that leveraged peer participants as human support [46-48] prioritized accountability and bonds within the peer cohort rather than expertise. An exception to this is the use of peer specialists, which uniquely applies both instrumental and relational factors. For example, Blonigen et al [44] used peer specialists who were professionally trained to use their lived experiences to support adherence to DHIs, leveraging expertise and benevolence. Finally, in most (14/22, 64%) studies, it was unclear whether expertise was conveyed to participants and, perhaps more importantly, whether the participants perceived their respective human support as experts and if such perceptions influenced DHI engagement and adherence.

People look for integrity, caring, and a sense of benevolence when determining the legitimacy of their coaches [31]. In our review, only a few (4/22, 18%) studies [54,60,65,67] proposed how the human support and participants establish trust (trustworthiness). For example, Borghouts et al [54] used “nurse promoteres,” who were trusted leaders in the local Spanish-speaking community, to provide support to participants. They built trust by connecting community members to health services and providing support that aligned with the intervention goals. Their existing role in the community made them an ideal source of human support in the intervention.

Other studies used different methods to establish and understand trust. Van Middelaar et al [67] intentionally designed the initial baseline consultation between participants and their assigned coaches to take place in person to better establish trust [67]. In the studies by Baron et al [76], Mohr et al [70], and Mohr et al [71], the first coaching session was an “engagement session,” designed to last longer than follow-up sessions to introduce the interventions, discuss participant goals, set expectations, and build rapport.

Although these studies discussed how they anticipated legitimacy, including trust, would be established, few assessed whether participants themselves perceived their human support as benevolent, trustworthy, and knowledgeable. On the other hand, Sayegh et al [65] and Wilhelmsen et al [60] assessed whether trust was established by interviewing participants about their experiences with the intervention, including their relationships with the human support. In both studies, participants expressed a sense of connection to their human support. For instance, participants suggested that their relationships with the coaches felt very personal and trusting [65] and that they could trust their therapists due to their expertise [60].

Although reciprocity was not explicitly mentioned in any of the articles, it may have been implied or inferred in some interventions. In Jesuthasan et al [24], coaches introduced themselves during the first week of the intervention and explained their roles, which may demonstrate reciprocity by framing the relationship in terms of the benefits that coaching may provide participants [31]. This is similarly evident in the studies by Baron et al [76], Mohr et al [70], and Mohr et al [71]. In the study by Lepore et al [72], an intervention study with low-income minority mothers who smoke, participants were explicitly made aware of the expectations regarding app usage and the role of the telephone counselors (ie, to uphold accountability and support participants throughout the duration of the intervention). This demonstrates a major hallmark of reciprocity, the patient-coach “contract,” in which both parties have clearly defined roles (eg, the patient is expected to log into the intervention and complete specific tasks or activities, while the coach provides time, attention, and support with problems that may arise) [31]. In a similar vein, although no study explicitly described benevolence, many suggested that benevolence was considered during intervention design and in defining the role of the human support. For example, human support was often described as “warm” and “friendly” (eg, [38]) and instructed to reflect a sense of caring through encouragement and affirmation [24,75]. Such direction appeared to be intended to ensure that participants perceive that their human support has their best interests in mind.

##### Bond

The notion of bond captures the emotional attachment between intervention participants and their human support, which may ultimately promote the effects of accountability [31]. The majority of studies that used SAM (14/22, 64%) did not explicitly mention methods or processes to encourage liking or bonding [31]. How interventions intended to cultivate bonds was briefly described by 8 studies [15,24,45-48,64,70,71]. Of these studies, 3 used social networking features, such as user profiles [46,47] and posting and commenting on users’ posts [48] to connect. In these studies, participants served as peer human support to one another. Duffecy et al [47] also suggested that the ability to create user profiles containing personal information allowed participants to increase group bonds. Meanwhile, Ho et al [48] suggested that commenting on each other’s posts created opportunities for off-topic discussions, thereby allowing participants to build emotional connections and trust with one another. In other studies, research assistants and facilitators received training on developing positive relationships and building trust to encourage user adherence to the intervention [15,24,64].

## Discussion

### Principal Findings

In this review, we found that the sources of human support could be categorized into the following: (1) peers and peer specialists, (2) health experts and practitioners, (3) trained coaches, and (4) members of the research team. Most individuals providing human support are paraprofessionals with no formal credentials nor professional training related to the intervention [79]. The advantage of using health experts and practitioners is that they are readily available since they are already embedded in the clinical setting. This can allow for a more seamless and streamlined implementation process. However, several system-level barriers must be considered. For example, providers may have concerns regarding the time and effort it takes to integrate interventions into their existing workflow [80], as their time and effort invested may not be reimbursed or compensated [81-84]. The use of paraprofessionals (eg, trained coaches) specific to the interventions could bridge this gap, as existing evidence suggests that interventions delivered by paraprofessionals are scalable, feasible, acceptable, and effective [85,86]. In addition, pairing paraprofessionals with DHIs can enhance treatment fidelity by minimizing human error or therapist drift, which is common in manualized treatment protocols [87]. Adequate training for paraprofessionals is key to ensuring fidelity and scalability. Rosenberg et al [79] offered an in-depth description of their coach training program based on learning theories and competency-based supervision.

One of the common sources of human support found in this review is peers and peer specialists. Peer-to-peer support could promote DHI engagement because their relationship is characterized by reciprocal accountability, wherein peers motivate each other to work toward their individual goals [19]. On the other hand, peer coaching models (eg, peer specialists) are frequently used to enhance intervention accessibility, engagement, and scalability by providing human support from coaches who share similar identities and lived experiences [88]. One common use of peer specialists can be found in recovery support for substance use [89], in which peers apply personal experience and knowledge to support individuals who are earlier in their recovery process [90,91]. Aligned with our review, Blonigen et al [44] used peer specialists to facilitate veteran participants’ engagement with a DHI for unhealthy alcohol use. Shared lived experiences give peer specialists credibility and offer participants a sense of belonging and hope for the future [19].

Although some studies have demonstrated the positive effects of these peer models [92-95], empirical support remains mixed [96-99]. These inconsistencies may be due, in part, to the lack of structured interventions, formalized training, and quality assurance mechanisms for peer specialists and other paraprofessional human supports [97,100]. A critical gap in the current literature is the lack of detailed descriptions of training protocols, core competencies, and supervision processes, which makes it difficult to assess fidelity, replicate successful interventions, or scale promising approaches. Developing and reporting standardized training protocols for human support providers in DHIs are essential to ensure consistency, maintain intervention quality, and enhance participant outcomes. This is especially important for peer specialists and other paraprofessional human support, who are not typically professionally trained for these specific roles and may require additional guidance. Such protocols should include clearly defined roles and responsibilities, communication strategies grounded in behavior change theory, ethical and boundary training, cultural competence, and ongoing supervision or fidelity monitoring.

Our findings suggest that the frequency and duration of human support interactions are closely tied to the mode of communication, with important implications for engagement and adherence in DHIs. This relationship aligns with the communication bandwidth principle proposed in SAM [31], which suggests that communication media differ in their ability to convey social presence, thus influencing the quality of the interaction and the strength of accountability. For example, phone and video calls—used in 64% of the interventions—typically involved less frequent but longer sessions, allowing for richer interpersonal exchanges that include verbal and, in the case of video, visual cues. These higher-bandwidth modes facilitate stronger perceptions of bond, legitimacy, and trust—core constructs of SAM—by supporting nuanced communication, emotional expression, and real-time responsiveness.

In contrast, text-based communication (also used in 64% of interventions) enabled more frequent but shorter and often asynchronous interactions. Although this lower-bandwidth mode may offer greater flexibility and accessibility, particularly for asynchronous support, it strips away nonverbal cues and could potentially diminish social presence. The findings imply that, although both modes have utility, higher-bandwidth communication may be more effective for establishing initial trust and accountability. In contrast, text-based support may be a more suitable supplementary strategy for maintaining ongoing engagement. Future DHI design should consider tailoring support frequency and duration based on the mode of communication and the complexity of the behavior change goals, striking a balance between efficiency and the need for relational depth.

The majority of studies (97%) used human support to promote accountability to improve engagement with DHIs, including monitoring users’ usage, sending reminders, checking in, setting goals, and discussing performance [101,102]. Each of these strategies may be positioned relative to each other on a spectrum based on the degree to which an approach is “active.” For example, using human support to monitor user performance alone is a passive approach as it does not necessarily promote engagement with DHIs. Performance monitoring is often coupled with more direct check-ins and reminders that allow participants to infer the social presence of human support. To improve engagement, human support can also set forth expectations by setting goals with users; for example, human support can help users set and review personal goals, as well as create plans for continued DHI use. When setting goals, the targets of the expectations can vary. In this review, most human support focused on process accountability*,* with a primary goal of increasing engagement with the DHI. On the other hand, Cheng et al [73] used automated support, quizzes, and email notifications to maintain accountability. Although the notifications were effective reminders to complete the weekly sessions and diary entries, quizzes prompted participants to pay more attention to the information as they promoted outcome accountability.

Although accountability was used across most studies, we did not see any use of instruments to measure accountability. Therefore, it is unknown whether the steps taken to ensure accountability had any effects, positive or negative, on accountability. Qualitative studies by Sayegh et al [65] and Wilhelmsen et al [60] interviewed participants about their experiences with the intervention. In each of these studies, participants described their experience with their human support, and their encounters resulted in different levels of accountability that promoted their use of DHIs. Parent participants in the study by Whiteside et al [61] also described observing that the interventions gave a sense of responsibility for their children’s symptom management. Meyerhoff et al [103] saw a need for supportive accountability measures for coached digital interventions and developed the Supportive Accountability Inventory (SAI). The SAI is a 6-item instrument assessing users’ perceptions of (1) whether they believe the coach pays attention to their use of DHIs and progress and (2) if their coach maintains expectations of user engagement with the DHIs and their effort toward treatment-related goals. Future studies should utilize validated measures such as the SAI [103] to assess the mechanisms by which accountability is achieved (eg, monitoring and expectation).

Additionally, many studies did not explicitly describe how bonds and legitimacy were used to cultivate accountability. Articles frequently used keywords such as connection, warmth, trust, and friendly, which may have been used to signal or allude to bonds (eg, [38,50,67]. However, without clear definitions or theoretical grounding, it becomes difficult to determine whether these references reflect bonds—which emphasize mutual liking, emotional connection, and respect (eg, therapeutic alliance) [104]—or relational legitimacy, which centers on perceptions of trustworthiness, benevolence, and expertise [77] and does not necessarily involve interpersonal liking [31]. One possible reason for this gap is that qualities associated with bonds or relational legitimacy are often assumed to be inherent in any supportive or therapeutic relationship. As such, these relational dynamics may be implicitly embedded in human-supported DHIs but are rarely made explicit in intervention design or measured in outcome evaluations.

To address this gap, future research should more deliberately integrate and measure these constructs using validated instruments. For example, the Working Alliance Inventory [105] could capture the strength of the bond, while the Credibility/Expectancy Questionnaire [106] may assess relational legitimacy and perceived coach expertise. Intervention protocols could also include structured relational strategies such as rapport-building scripts, credibility-enhancing coach introductions, and personalized messaging that reinforces benevolence and competence. Last, qualitative evaluations are essential for understanding how participants perceive relational legitimacy, which, in turn, influences their behaviors. Although many interventions aim to build legitimacy, few assess how it is experienced. Interviews and open-ended feedback can capture these relational nuances, offering critical insight into how legitimacy fosters engagement and adherence in DHIs.

In this scoping review, we found certain studies used human support as the main deliverers of the intervention content, corresponding to Types 1 and 2 in the taxonomy in Pineda et al [39]. As aforementioned, SAM was proposed to suggest the specific use of *coaches* to support user engagement and adherence to DHIs [31]. This aligns with Type 3 within the taxonomy. The tension among Types 1, 2, and 3 within this scoping review indicates variations in the application of SAM to DHIs. These studies did not appear to utilize SAM in the way that it was originally intended. As such, it is necessary to clearly define intervention goals and subsequent roles of coaches when considering the use of human support in DHIs.

We found that only 6 (17%) of the 36 studies focused on a youth population, yet youth are increasingly intertwined with technology, and many seek and share health advice through social media [107,108]. One study [61] targeted children with anxiety, but the intervention itself was designed for both parents and their children, some as young as 8 years old. In our review, we found that the use of peers (eg, [46-48,50]) is a common strategy that aligns with the social affordances described by Wong et al [109]. Since youth, adolescents, and young adults are particularly interested in and influenced by their peers, future studies may consider leveraging peer influence in accordance with SAM to enhance DHI engagement and adherence among younger populations. For example, adolescents and young adults may be motivated to adopt DHIs that incorporate social components, as these may foster social support and a sense of belonging [110,111].

### Limitations

To our knowledge, this is the first scoping review to explore the application of human support strategies in DHI design to promote engagement. Several potential limitations should be considered. First, there is a chance that we missed relevant articles, as this review only included peer-reviewed studies and excluded gray literature. Another limitation is that, although this review focused on the 3 core factors (ie, accountability, bonds, and legitimacy) of SAM, the model has additional components that inform the pathways to adherence (eg, motivation and communication bandwidth). They are also important factors that can enhance the influence of accountability on adherence. Last, our search terms were designed to narrowly capture literature that is more likely to apply SAM. It is possible, however, that other studies utilize human support but do not term it as such. Authors may use different keywords that were not captured by our search terms. We attempted to address this through additional hand-searching.

### Conclusion

Strategies to improve engagement with DHIs are undoubtedly needed. This scoping review examines the current state of knowledge regarding the strategy of human support and the application of SAM in DHIs. Our review identified inconsistencies in the application of SAM across studies. For example, although SAM focuses on using human support primarily to improve DHI engagement and adherence, studies used human support for the administration of interventions. Further, bond and legitimacy, major constructs that, according to SAM, promote accountability, were rarely explicitly considered and discussed by authors. Further research should demonstrate more intentional application of these constructs, documenting how interventions were designed to promote bonds and establish legitimacy and measuring each with psychometrically sound instruments. Studies should also examine communication bandwidth and motivation as potential moderators of adherence [31]. For example, we found that interaction frequency and duration appeared to be influenced by the type of communication (eg, call, text, videoconferencing). Future studies could focus on the frequency and duration (ie, bandwidth) of these communications, as well as their quality, assuming that greater communication could lead to improved task completion, enhanced interpersonal relations, and greater social presence [31]. Similarly, it is worth exploring how human support can move users from operating solely on extrinsic motivation to intrinsic motivation as well. Techniques such as motivational interviewing may facilitate this transition. Last, future studies may consider comparing and evaluating the impact of human support strategies on engagement and adherence. We suspect that those strategies that are highly personalized and tailored to the target population (ie, appropriate for age or developmental stage, symptoms or health conditions, mode of delivery) will lead to better engagement and adherence. For example, utilizing peers as a form of human support is acceptable, appropriate, and potentially effective among youth or young adults. In sum, adherence and engagement to DHIs have real-world implications, and this review sheds light on how human support is currently utilized to promote engagement. As the field continues to respond to the ubiquitous nature of technology within our society, the prospect of DHIs offers accessibility and scalability for many populations challenged by a myriad of health conditions. However, the efficacy of DHIs cannot be realized in the face of the law of attrition. We thus require thoughtful, intentional, and faithful integration of human support to optimize DHI engagement and adherence.

## Appendix

Multimedia Appendix 1 Summary of research studies.

Multimedia Appendix 2 Incorporation of supportive accountability model (SAM) components in digital health intervention (DHI) designs.

Multimedia Appendix 3 PRISMA-ScR Checklist.

## Abbreviations

DHI: digital health intervention
DMHI: digital mental health intervention
mHealth: mobile health
PRISMA-ScR: Preferred Reporting Items for Systematic Reviews and Meta-Analyses Extension for Scoping Reviews
SAI: Supportive Accountability Inventory
SAM: supportive accountability model
SCT: social cognitive theory
SDT: self-determination theory

## Data Availability

The data that support the findings of this study are available from the corresponding author upon reasonable request.
