# Supplementary material for: Application of the Supportive Accountability Model in Digital Health Interventions: Scoping Review
Source: J Med Internet Res. 2025 Sep 26;27:e72639. doi: 10.2196/72639 (PMC12514419; doi:10.2196/72639)
Supplement: Multimedia Appendix 1 [file jmir_v27i1e72639_app1.docx]

| **Reference** | **Study Design** | **Target of Intervention** | **Study Population Age** | **Engagement Model** | **Engagement Measure** | **Human Support Strategy** | **Findings Related to Engagement** |
| --- | --- | --- | --- | --- | --- | --- | --- |
| Baron et al. (2018) | Mixed (survey with quantitative measures and open-ended questions) | Sleep duration | Adults (18-65) | SAM | Completion percentage of coaching sessions and sleep diary days; percentage of nights FitBit worn | Initial 20 min. "engagement" session, followed by weekly 10 min. telephone coaching sessions, with options for support via email/text as alternatives to telephone sessions | Participants completed 100% of coaching sessions and 50-60% of sleep diary days. They wore Fitbit 50-80% of nights. |
| Berman et al. (2018) | Quantitative (nonblinded, single-arm) | Type 2 diabetes | Adults (18+) | None identified | Average number of recorded app actions per day (e.g., planning/reporting meals, building shopping lists) | Health coaching phone calls every 2 weeks | N/A  Findings focused on target outcome (i.e., hemoglobin and medication use). No comparison group with participants who received the intervention without health coaching calls. |
| Blonigen et al. (2021) | Quantitative (single-arm, pre/post) | Positive screen for problem drinking on AUDIT-C and who had not received treatment following positive screen. | Adults (18+) | SAM | Time spent in app, number of days used, number of daily and weekly interviews completed | Weekly 15-30 min. phone sessions with peer specialists | N/A   The intervention was found to be highly acceptable to study participants, but there was no comparison group with participants who received the intervention without support from peer specialists. |
| Borghouts et al. (2022) | Mixed (4 surveys and interview) | Isolated/loneliness | Older adults (60+) | None identified | Self-reported app use and intention to use the app | Nurse promoteres (volunteer nurse interns and promoteres that work with the Spanish-speaking community in the county) | N/A   19 out of 26 participants reported feeling more confident about using technology (including the intervention) following training from the promoteres. None described the impact of promoteres' on their engagement with the intervention itself. |
| Carpenter et al. (2022) | Quantitative (secondary analysis of randomized pilot study) | Overweight or obese, according to BMI | Adults (18+) | SAM | Defined relative to "disengagement" (i.e., the point at which participant failed to self-monitor consistently in a period of 2 weeks.   "Re-engagement" also measured - any point at which participant self-monitored consistently after disengagement. | Trained behavioral weight loss interventionist | Participants who received phone calls consistently self-monitored their dietary intake for a greater number of weeks than those who did not receive the phone calls (p=.006). No difference for self-monitoring weight and physical activity.   Among participants who disengaged from self-monitoring physical activity, those in the phone call intervention group re-engaged in self-monitoring in fewer weeks (i.e., sooner) than those who disengaged in the group with no phone calls. No difference in re-engagement for self-monitoring weight and dietary intake. |
| Cheng et al. (2023) | Mixed (survey with quantitative measures and open-ended questions) | Insomnia | Adults (18+) | None identified | One of the themes generated in open-ended responses was "digital person-to-person component," where participants talked about their experiences with the app's features meant to increase feelings of interpersonal connection. | Animated and interactive digital "therapist"  Human support from the research team for "research-related processes" (e.g., signing up for the app) | Overall, participants thought that the animated therapist entertaining and fun, which may suggest success in promoting engagement. Some preferred a human support instead. |
| Chew et al. (2021) | Quantitative (single-arm, prospective) | Overweight or obese, according to BMI | Adolescents (10-16) | SAM | Weekly frequency of information entered for meal consumption, body weight, physical activity, and coaching sessions | Weekly 15-min. individual coaching via video chat, phone, or text with health coaches.  In between coaching sessions, coaches may be contacted through short message service text messages, email, or in-app messaging function.   Other strategies include supplemental materials: e-workbook, biweekly email newsletter, physical activity demonstration videos, blog posts, downloadable healthy eating cookbooks. (From Cueto et al., 2019) | N/A   Participants completed a median of 7 weeks out of the 12-week intervention. No comparison group that completed the intervention without a coach. |
| Cueto et al. (2019) | Quantitative (retrospective cohort study) | Obese or overweight, according to BMI | Youth (5-18) | SAM | Total number of health coaching sessions received | Weekly 15-min. individual coaching via video chat, phone, or text with health coaches.  In between coaching sessions, coaches may be contacted through short message service text messages, email, or in-app messaging function.   Other strategies include supplemental materials: e-workbook, biweekly email newsletter, physical activity demonstration videos, blog posts, downloadable healthy eating cookbooks. (From previous paper) | N/A   Retention was high -- almost 80% of the participants engaged with the program for the duration of their commitment period (4 weeks, 12-16 weeks, or 24 weeks). This study did not have a comparison group. |
| Chhabria et al. (2020) | Quantitative (psychometric validation and randomized trial for intervention) | Overweight or obese, according to BMI | Adults (18+) | SAM | Platform use and exercise completion (e.g., use of self-monitoring strategies) | 10-15 min. phone calls (8 weekly, 4 biweekly, and 2 monthly contacts)  Intervention was administered by trained interventionists (either a clinical psychologist or dietitian, both experienced in delivering behavioral weight management programs). | Group with phone-based support had greater SAM total scores and subscores for Support for Healthy Eating Habits and Perceptions of Accountability. Higher SAM scores were associated with greater adherence. |
| Dennison et al. (2014) | Quantitative (2-armed randomized trial) | Healthy to obese, according to BMI | Adults (18+) | SAM | Number of intervention sessions completed in an 8-week period | 10-min. phone calls with trained coaches (post-grad students and research assistants affiliated with university health psychology research center). Calls scheduled for after Weeks 1 and 4. | Intervention use in both arms (with and without the coaching calls) was low. 1/3 of participants didn't complete a single session, while most who completed any sessions only did one or two.   Coaching uptake was low: 58% of participants in coaching arm did not receive coaching calls. Of the 104 participants that had coaching, most (n = 58, 55.8%) had just one call. Only 46 participants (18.6%, 46/247) received both calls (i.e., a full dose of coaching). |
| Duffecy et al. (2013) | Quantitative (2-arm RCT) | Depressive symptoms | Adult cancer survivors (19+) | SAM | Number of logins | Internet support group (ISG) with other intervention participants; interactions took place via discussion board | Trend-level significance (P = .15) was achieved in the whole sample, with greater average logins in the ISG group vs. the non-ISG group. |
| Duffecy et al. (2019) | Quantitative (feasibility RCT) | Pregnant (between 20 and 28 weeks gestation at baseline assessment) and had mild-moderate depressive symptoms (PHQ-8 screener) | Adults (18+) | None identified | Total number of log-ins, completion of tools, and lessons | Human support via "share" features (i.e., likes, comments, nudges, and posts) | Adherence was the same between both the control group and the group with "share" features, but uptake of the peer network features was low.   59% (10/17) of the participants used at least one feature, most commonly the ability to comment on discussion questions or posts. Initiating status updates also was common (53%). The ability to like status updates and nudge group members were utilized less (35% and 12%, respectively). |
| Duffecy et al. (2022) | Quantitative (2-arm RCT) | Pregnant (between 20- and 28-weeks gestation at baseline assessment) and had PHQ-8 score 5-14 | Adults (18+) | SAM | Primary marker was number of logins. Additional measurements included: time spent on site, number of lessons viewed, and tools used (e.g., number of thoughts entered, number of relaxation modules accessed, number of emotions rated, etc.) | Peer support through collaboration on the “Activity Feed," a newsfeed that updated with participants' completed activities on the site and allowed all group members to post, “like”, and comment or provide feedback on other people’s posts. | No significant difference on primary adherence measure (number of logins) between the control and the peer support group. Control group entered more thoughts than the peer support group (*P* = .035). |
| Fletcher et al. (2019) | Mixed (quantitative measures and interviews) | Trauma-exposed veterans | Adults (18+) | None identified* | N/A | 50-minute coaching sessions via video teleconferencing after the initial module and every 2 completed modules afterwards. Coaches could access a program dashboard that allowed them to view participants' most recent login and total number of minutes spent per module. Participants could correspond with coaches coach between sessions over phone or through secure messaging platform. |  |
| Glasgow et al. (2011) | Quantitative (3-arm RCT) | Type 2 diabetes | Adults (18+) | None identified | Website visits | Two 10-min. follow-up calls at Week 2 (with study team member) and Week 8 (with diabetes care manager).   Group session, led by nutritionist, before 4-month assessment. | Participants in the human support condition have statistically significant greater website use than participants without human support. Specifically, participants in the condition with human support engaged in self-monitoring of medication adherence more than those in the condition without human support. |
| Ho et al. (2016) | Mixed | Exclusions: severe depression based on CESD scale, diagnosed with medical condition which makes participation dangerous/inappropriate, prescribed antidepressant medication, suicidal | Adolescents (14-19) | SAM | Website use (e.g., logins, time spent on site, days used site, use of intervention tools) | Peer networking capabilities on a website | Positive correlation between "exchange comments" (i.e., comments that were posted as an exchange between two or more participants) and mean time spent on the website. |
| Jesuthasan et al. (2022) | Quantitative (2-arm) | Employees from a company in Malaysia | Adults (18+) | SAM | Retention, rate of feature exploration (i.e., engaging with the feature at least once), total number of messages sent to coaches, number of modules completed | Prompts from an intervention "assistant" (whose roles is to provide practical support with accessing and using the digital intervention).   Coaching available from health professionals (who administer parts of the intervention and provide personalized therapeutic content). | No difference in activity between group with the assistant and group without. |
| Kar et al. (2020) | Quantitative | Type 2 diabetes and BMI > 29kg/m2 | Adults (18+) | None identified* | Interactions within the 3 components of the app:  Learn: number of articles read  Track: number of times a participant registered or viewed weight or steps reading  Support: number of messages sent/read in private or group chat channels. | Health coaches (registered dietician or nutritionist) available for personalized support via text. Peer support group also available among participants. | N/A   Engagement was related to better health outcomes. There was no comparison group that completed the intervention without the human support component. |
| Kelders et al. (2015) | Quantitative (8-arm) | Mild to moderate depression symptoms, according to CESD | Adults (18+) | None identified | Log files (reaching Lesson 9 is considered adherent) | Text messaging coaching written by researchers prior to study. Each week, three text messages containing motivational mindfulness, and content-related information were sent to participants. | No difference found between human support condition and the automated form of support. |
| Lattie et al. (2017) | Mixed (interviews and quantitative survey) | Adolescent depression symptoms | Adolescents (14-19 years) | None identified* | Program usage (system logins, lessons read, use of different platform tools such as mood rating) | Two human-supported conditions tested:  (1) Peer-led intervention (2) Clinician-led intervention  Participants can access Activity Feed on homepage and engage in social networking platform feature (e.g., commenting, likes and nudges). Feed updates with participants' completed activities. Peer/clinician guide and participants could nudge users when they were inactive. | No statistically significant difference in tool usage between peer-led and clinician-led group. No control condition to identify whether intervention effectively reduces depressive symptoms among adolescents. |
| Lederman et al. (2014) | Qualitative | Experience of a first episode of a psychotic disorder | Youth (15-25) | SAM | N/A | Peers and moderators via social networking features (e.g., posting and commenting on other participants' posts).   Direct messaging enabled between participants and moderators | Users felt a sense of belonging and identification with others. The intervention engendered accountability. |
| Lepore et al. (2021) | Quantitative | Smoking mothers with children <6 years old | Adults (18+) | SAM | Number of days the app was used | Up to 5 phone counseling sessions over 3 months | Counselor monitoring and supportive advice positively associated with app usage. Counselor support also increased app engagement more among smokers who were not planning to quit than among those who were already planning to quit. |
| Mira et al. (2017) | Quantitative | Experiencing at least one stressful event and depressive symptoms, according to BDI-II | Adults (18+) | None identified | Intervention completion | Compared 2 support modes:  (1) Automated support (two weekly automated phone messages encouraging participants and reminding them of importance of completing intervention tasks); and  (2) Human support via two weekly supportive calls from a therapist offering motivation/positive support (non-clinical) | A non-statistically significant higher percentage of the human support group completed the intervention modules. |
| Mohr et al. (2013) | Quantitative (2-arm RCT) | Major depressive disorder | Adults (18+) | SAM | Number of days logged in, number of days until last login, number of times lessons were viewed, number of times tools were used, and the number of different types of tools used, number of completed coaching sessions | Initial 30-45 min. "engagement session" with coach, followed by weekly 5-10 min. telephone-based coaching sessions. Participants could text/email coaches in between sessions. | Coached participants had significantly greater logins (*P* = .01), used the intervention for longer (*P* = .007), viewed more lessons (*P* = .03) and used a greater variety of tools (*P* = .02) compared to the non-coached participants. |
| Mohr et al. (2019) | Quantitative (4-arm RCT) | Depression (PHQ-8) and anxiety (GAD-7) | Adults (18+) | SAM | Time to last use, number of app sessions, and number of apps downloaded | Tested 4 conditions:  (1) Coached with automatic recommendation notifications (2) Coached without recommendations (3) Self-guided with recommendations (4) Self-guided without recommendations  Coached groups received initial 30-45 min. "engagement" phone call with coach, followed by 2-3 texts weekly. Participants could text coaches as well. | No significant differences in time to last use between coached groups and self-guided groups. No significant effect of coaching on number of apps sessions. Coached groups downloaded more apps than self-guided groups. (Wilcoxon *P* < .001). |
| Possemato et al. (2022) | Quantitative (3-arm RCT) | Veterans with unmet mental health needs | Adults (18+) | SAM | Number of logins | Tested 3 conditions:   (1) Control (waitlist) (2) Self-directed intervention (3) Peer-supported intervention  20-minute peer support sessions delivered over phone 5 times over the course of the 8-week intervention. Peer specialists aimed to promote participants' engagement in the intervention and application to their daily lives. Peer specialists were encouraged to share their own experiences of overcoming life’s problems, as appropriate. | Participants in peer support group had significantly more logins than did self-directed participants. |
| Renfrew, Morton, Morton, Hinze, Beamish, et al. (2020) | Quantitative (3-arm RCT) | Eligibility and exclusion criteria not reported | Eligibility and exclusion criteria not reported | SAM | Total number of weekly videos viewed, daily and weekly challenge activities completed (based on points awarded -- 1000 points possible; attending video-conference call in that condition was awarded 10 points) | Compared 3 support modes:  (1) Automated emails,  (2) Personalized SMS messaging, and  (3) Facilitated videoconferencing (weekly, 20-30 minutes, led by post-grad in lifestyle medicine).   Weekly Zoom call included recap of weekly content, sharing new learnings and challenges, and discussion on how to incorporate strategies into daily life. | Adherence was not significantly different between groups. |
| Renfrew, Morton, et al. (2020b) | Quantitative (3-arm RCT) | Eligibility and exclusion criteria not reported | Eligibility and exclusion criteria not reported | SAM | Total number of videos viewed, daily and weekly challenge activities completed (based on points awarded -- 1000 points possible; attending video-conference call in that condition was awarded 10 points) | Compared 3 support modes:  (1) Automated emails,  (2) Personalized SMS messaging, and  (3) Facilitated videoconferencing (weekly, 20-30 minutes, led by post-grad in lifestyle medicine).   Weekly Zoom call included recap of weekly content, sharing new learnings and challenges, and discussion on how to incorporate strategies into daily life. | Adherence was not significantly between participants who received their preferred support strategy and those who did not. |
| Sayegh et al. (2024) | Qualitative (semi-structured interviews) | Sickle cell disease, solid organ transplants, or type 2 diabetes, taking at least one daily oral medication | Youth (15-20) | SAM | N/A | Compared 3 modes of support:   (1) 5-min. coaching calls between 3 and 5 times a week; (2) 5-min. coaching text message interactions between 3 and 5 times a week; and (3) Daily automated text medication reminders requesting a text back to confirm receipt. | Coaching helped the youth develop routines and strategies. Phone calls allowed for the development of more personal connections; youth were motivated to avoid disappointing their coaches.   Calls were sometimes seen as disruptive to daily life, whereas texts were flexible and therefore more convenient.   Some participants felt that automatic text reminders were helpful. Those who had human coaches described that automated support would have been somewhat uncomfortable. |
| Stiles-Shields et al. (2019) | Quantitative (3-arm) | Depression (PHQ-8 and QIDS) | Adults (18+) | SAM | App "launches", number of events logged, number of thoughts logged, number of event reviews, and number of event reviews. | Weekly 5-min. coaching calls (email if participant could not be reached) with purpose of promoting engagement with the intervention. | Study compared two different interventions which utilized coaching. No control groups were used to identify whether coaching had an effect on adherence/engagement compared to a self-guided intervention condition. |
| Smart et al. (2022) | Qualitative (qualitative summative evaluation and semi-structured interviews) | Overweight, according to BMI | Adults (21-65) | None identified | Total number of weeks (out of 8) that a text message goal was set; number of texts sent and received by participant | Text messaging-based coaching | Health coaches were helpful in making participants feel more accountable to the goals that they set for themselves. Health coaches also provided technical support for using FitBits. |
| Tomasino et al. (2017) | Quantitative (3-arm RCT) | Older adults with depression (PHQ-8 or Geriatric Depression Scale-15) | Adults (65+) | None identified* | "Use" was measured via sessions, lesson completion, and peer support features utilized. A session was defined as a sequence of user-initiated actions separated by less than 10 minutes between events. | Three conditions tested: (1) Control (waitlist) (2) Individual intervention (3) Intervention w/ peer support  Peer support condition utilized social networking platform features (user profiles, “activity feed”, “status” posts, and participant-selected tool content). Participants interacted via "like", "comment", and "nudge" features. "Nudging" a participant sent an automated email reminder to return to the intervention platform.   Coaches had access to a dashboard, where they could view participant activity and responses to weekly symptom questionnaires, as well as send and receive messages to and from participants. Coaches had weekly 10-15 min. phone calls or messages with participants to encourage use and reinforce progress, answer questions, and support skill application, among others. | Differences between the peer-support group and non-peer support group for number of sessions and average number of lessons completed were not statistically significant (*P* > .50).   Weekly sessions and lesson completion between the two groups also yielded non-statistically significant differences (all *P* values > .10). |
| van Middelaar et al. (2018) | Qualitative (semi-structured interviews) | Adults with increased risk of cardiovascular disease | Older adults (65+) | SAM | N/A | Coaching | Participants were willing to talk about health behaviors and potential lifestyle goals after building trust with the coach. Trust was important to initiate and sustain platform use. |
| Whiteside et al. (2019) | Mixed (quantitative measures and interviews) | Children with anxiety disorders and their parents | Children (under 18) and their parents | SAM | N/A | Weekly sessions with therapist who provides psychoeducation and discusses with family how to apply the psychoeducational material to their child's symptoms. Therapist guides family on facilitating exposures for child. Exposures by therapist or family are recorded in the "check-up" module of the platform. Therapist is able to monitor the check-up page in the web-based portal and provide direction in-session and remotely throughout the week. | Interview data indicated that parents and patients believed the application was effective at keeping them accountable to engaging in exposures in between therapy sessions. |
| Wilhelmsen et al. (2013) | Qualitative (semi-structured interviews) | Mild to moderate symptoms of depression | Adults (18-65) | None identified | N/A | Face-to-face 20-30 min. "consultations" with therapist.   Consultations limited to the following subjects: symptom monitoring, previous module in the intervention, and introducing next module and discussing participant's motivation. | Consultations with therapist were "helpful and motivating." Connection with therapist was established via understanding, flexibility, acknowledgement and openness. Expert status of therapist generated confidence and trust in their feedback. The time constraint (20-30 minutes) was stressful to participants since they wanted to discuss their challenges in more depth (as in traditional psychotherapy). |
| Yardley et al. (2014) | Quantitative (randomized 4-arm parallel non-blinded trial) | BMI >= 30 (or >= 28 with hypertension, hypercholesteremia, or diabetes) | Adults (18+) | None identified | Number and format of nurse support sessions attended | Compared 4 modes of support:   (1) Usual care,  (2) Web-based intervention only, (3) Web-based intervention with basic nurse support (3 sessions in 3 months), and (4) Web-based intervention with regular nurse support (7 sessions in 6 months) | Session completion rates were very similar across all intervention groups. Attrition higher than comparable interventions with no health professional contact. |

Studies that do not identify a specific engagement model but cite Mohr et al (2011) are noted by *.

**References**

Mohr DC, Cuijpers P, Lehman K. Supportive accountability: a model for providing human support to enhance adherence to eHealth interventions. J Med Internet Res 2011 Mar 10; 13(1):e30. doi: 10.2196/jmir.1602
