# Supplementary material for: Application of the Supportive Accountability Model in Digital Health Interventions: Scoping Review
Source: J Med Internet Res. 2025 Sep 26;27:e72639. doi: 10.2196/72639 (PMC12514419; doi:10.2196/72639)
Supplement: Multimedia Appendix 2 [file jmir_v27i1e72639_app2.docx]

| **Reference** | **Human Support Strategy** | **Bond (i.e., "liking")** | **Accountability (i.e., "the implicit or explicit expectation that an individual may be called upon to justify his or her actions or inactions")** | **Legitimacy (i.e., expertise, reciprocity, trust, and benevolence)** |
| --- | --- | --- | --- | --- |
| Baron et al. (2018) | Initial 20 min. "engagement" session, followed by weekly 10 min. telephone coaching sessions, with options for support via email/text as alternatives to telephone sessions | Not mentioned | The goal of the coaches' dashboard, where coaches could view participants' intervention engagement and data, was to increase supportive accountability. They also discussed participants' data during weekly coaching sessions. | The initial engagement session included coach introductions, clarifying roles of the coach, and the participants’ goals for the program (reciprocity). |
| Blonigen et al. (2021) | Weekly 15-30 min. phone sessions with peer specialists | Not mentioned | Peer support calls were used to ask about app use, provide suggestions on how app content could be applied to reach participants' drinking goals, offer technical support to navigate the app, and encourage ongoing app use and suggest action plans tailored to participant needs. | Peer coaches were in recovery and were encouraged to share their lived experience of substance use problems (expertise, trust, and benevolence). |
| Carpenter et al. (2022) | Human support via "trained behavioral weight loss interventionist" | Not mentioned | Calls began with a check-in on self-monitoring behaviors (frequency) and progress towards goals since the previous call. | The behavioral weight loss interventionist was trained, but it is unclear whether legitimacy is established with the participants. |
| Chew et al., (2021) | Weekly 15-min. individual coaching via video chat, phone, or text with health coaches.  In between coaching sessions, coaches may be contacted through short message service text messages, email, or in-app messaging function.   Other strategies include supplemental materials: e-workbook, biweekly email newsletter, physical activity demonstration videos, blog posts, downloadable healthy eating cookbooks. (From Cueto et al., 2019) | Not mentioned | At each weekly check-in, coaches "monitored" participants' self-reporting (weight, physical activity, dietary behaviors). After the check-in, coaches email participants with a summary of their call and a tailored plan for the next week. | The coaches were certified by Kurbo, but it is unknown whether legitimacy was established with the adolescent participants. |
| Cueto et al. (2019) | Weekly 15-min. individual coaching via video chat, phone, or text with health coaches.  In between coaching sessions, coaches may be contacted through short message service text messages, email, or in-app messeging function.   Other strategies include supplemental materials: e-workbook, biweekly email newsletter, physical activity demonstration videos, blog posts, downloadable healthy eating cookbooks. (From previous paper) | Not mentioned | At each weekly check-in, coaches "monitored" participants' self-reporting (weight, physical activity, dietary behaviors). | The coaches were certified by Kurbo, but it is unknown whether their expertise was communicated with the adolescent participants. Further, this particular study identified that summary emails following check-ins contained praise for the participants' goals met from the previous week (benevolence). |
| Chhabria et al. (2020 | 10-15 min. phone calls (8 weekly, 4 biweekly, and 2 monthly contacts)  Intervention was administered by trained interventionists (either a clinical psychologist or dietitian, both experienced in delivering behavioral weight management programs). | Not mentioned | The SAM measurement instrument asks about participants' perceptions of accountability to others but it does not specifically ask about whether participants felt accountable to the coach in the intervention. | Phone-based support came from either a clinical psychologist or dietitian, both experienced in delivering behavioral weight management programs. Nothing reported about whether attempts to establish legitimacy took place. |
| Dennison et al. (2014) | 10-min. phone calls with trained coaches (post-grad students and research assistants affiliated with university health psychology research center). Calls scheduled for after first and fourth weeks. | Coaches were encouraged to be "warm and friendly". | Coaches monitored usage and gave feedback on progress. | Coaches offered support and encouragement for use of the website (benevolence). Other aspects of legitimacy were not addressed. |
| Duffecy et al. (2012) | Internet support group (ISG) with other intervention participants; interactions took place via discussion board | Discussion board allowed participants to build relationships with one another. | Initial staff phone call and first lesson clarified expectation for frequency of logins. Time since last login could be viewed by all participants to encourage group accountability. Participants could "buzz" each other, sending a reminder email to individuals to return to the platform. | Participants were encouraged to create profile sharing details about their cancer experience (personal credibility, i.e., expertise/trust). |
| Duffecy et al. (2022) | Peer support through collaboration on the “Activity Feed," a newsfeed that updated with participants' completed activities on the site and allowed all group members to post, “like”, and comment or provide feedback on other people’s posts. | Participants were invited to create their own individual profiles to increase group bond. Participants were encouraged to share enough to get to know each other, but not to share identifying information due to potentia loss of privacy. | Participants had access to an activity feed which updated with participants' completed activities. Group members could "like", comment, and post. Participants' profiles were linked to “Personal Garden Plots” and a “Community Garden Plot.” Completion of intervention tasks and acitivities added garden gnomes and flower collections to the garden plots. These served as a visual representation site use and increased accountability to one another to complete activities. | Not mentioned |
| Ho et al. (2016) | Peer networking on a website | Bond is promoted through the social features (e.g., media sharing, intervention activity, commenting). The commenting feature supports off-topic discussions "that are required to build emotional connection and trust." | Expectations of participants are made explicit, and peer network members' activities can be viewed by everyone, allowing each member to hold one another accountable. Members tiles (profiles) are color-coded according to whether their login expectations are being met. Those who haven't logged in for more than 4 days have profiles that are colored brown, instead of blue, and peers can click on a button to send a supportive email to the participant, encouraging them to return to the website. | Since the human supports are other peers participating in the intervention, expertise may not be established. Reciprocity, trust, and benevolence were not discussed but may be present given the nature of peer relationships. |
| Jesuthasan et al. (2022) | Prompts from an intervention "assistant" (whose roles is to provide practical support with accessing and using the digital intervention).   Coaching available from health professionals (who administer parts of the intervention and provide personalized therapeutic content). | The assistant aimed to develop bond with participants via regular messaging, including personalized messages that followed up on participants' in-app activity. | The assistant helped set up accountability structures, prompted participants to engage in activities, and monitored their app use. | Coaching team had various credentials and came from many different disciplines. They all had the necessary credentials to be able to practice in their respective fields. It is unclear whether their expertise was established with participants. Coaches introduced themselves in the first week, explaining their roles as a coach (reciprocity). Trust and benevolence were not explicitly mentioned with respect to the coaches. The assistant encouraged participants by congratulating them when they completed activities (benevolence). Expertise of the assistant was not explicitly reported but may have been linked to their employment with the app developers. Building trust may have been encouraged via bond. |
| Mohr et al. (2013) | Initial 30-45 min. "engagement session" with coach, followed by weekly 5-10 min. telephone-based coaching sessions. Participants could text/email coaches in between sessions. | In the initial 30-45 min. engagement session, coaches "established a bond." No further detail. | Coaching sessions included positive reinforcement of login and site use and encouraged use of the intervention when login goals were not met. | Coaches were to "convey benevolence and expertise and discuss treatment expectations" (reciprocity) during the engagement session. Coaching focused on establishing a supportive relationship (benevolence), setting and reviewing login goals (reciprocity), and answering any questions regarding the functionality of the site (expertise). |
| Mohr et al. (2019) | Initial 30-45 min. "engagement" phone call with coach, followed by 2-3 texts weekly. Participants could text coaches as well. | One of the goals of the initial engagement call was to build rapport. | Coaches texted participants 2-3 times a week to provide support and encouragement, reinforce app use, and check-in on progress/challenges. | In the initial engagement call, coaches explained the program, discussed participant goals for symptom management, and set expectations for the coach-participant relationship (reciprocity). They also ensured that the intervention was properly installed on the participants' phones (expertise).  Coaches at minimum had an undergraduate degree in psychology (or related field) and were trained and monitored by one of the coaching manual authors. Authors do not state whether this was communicated to participants to further establish expertise. |
| Lederman et al. (2014) | From peers and moderators via social networking features (e.g., posting and commenting on other participants' posts). Direct messaging enabled between participants and moderators | Moderators were instructed to: encourage a tone of hope and optimism, create their own profiles to present as real people and more relatable to users. | Accountability was applied through the use of the newsfeed (users could see each other's activity). Moderators were also able to view users' metadata (e.g., logins over time, module pages read, posts per user, etc.) | Expertise is assumed by other users (i.e., other patients/clients), who share the same illness or experience. Moderators were clinic staff. |
| Lepore et al. (2021) | Up to 5 telephone counseling sessions over 3 months | Not mentioned | Participants were told that telephone counselors would routinely monitor app entries to learn more about their smoking habits and to guide their advice about change efforts. | Participants were told that the telephone counselor would remind them or initiate troubleshooting when app use adherence was low. Participants were aware of the expectations, monitoring, and accountability related to app usage from the beginning of the intervention (reciprocity). |
| Possemato et al. (2022) | Tested 3 conditions:   (1) Control (waitlist) (2) Self-directed intervention (3) Peer-supported intervention  20-minute peer support sessions delivered over phone 5 times over the course of the 8-week intervention. | Peer specialists were encouraged to share their own experiences of overcoming life’s problems, as appropriate. | Peer specialists aimed to promote participants' engagement in the intervention and application to their daily lives. | Expertise is assumed by other users (i.e., other patients/clients), who share the same illness or experience. Moderators were clinic staff. Extent to which these trainings were communicated to participants to convey legitimacy is not described. |
| Renfrew, Morton, Morton, Hinze, Beamish, et al., 2020 | Compared 3 support modes:  (1) Automated emails,  (2) Personalized SMS messaging, and  (3) Facilitated videoconferencing (weekly, 20-30 minutes, led by post-grad in lifestyle medicine).   Weekly Zoom call included recap of weekly content, sharing new learnings and challenges, and discussion on how to incorporate strategies into daily life. | It is suggested that video conferencing, which most closely replicates face-to-face settings, accessibly allows participants to bond with each other in a virtual group setting. | Text message supports focused on accountability to process rather than outcome (i.e., completing target behaviors). Zoom group support included room for discussion on participants' new learnings, challenges, and how they were applying learned strategies to their lives. | The facilitator had a post-graduate degree in lifestyle medicine and experience hosting preventative health videoconferences for participant. Prior training included 6 mentoring sessions on how to facilitate videoconferences. It is not mentioned whether and how the facilitator established legitimacy with the participants. |
| Renfrew, Morton, et al. (2020b) | Compared 3 support modes:  (1) Automated emails,  (2) Personalized SMS messaging, and  (3) Facilitated videoconferencing (weekly, 20-30 minutes, led by post-grad in lifestyle medicine).   Weekly Zoom call included recap of weekly content, sharing new learnings and challenges, and discussion on how to incorporate strategies into daily life. | Videoconferencing is stated to allow opportunity to develop bond as a key tenet of supportive accountability. | Modes of accountability not discussed. | Videoconferencing is stated to allow opportunity to develop legitimacy as a key tenet of supportive accountability. |
| Sayegh et al. (2024) | Compared 3 modes of support: 5-minute coaching calls between 3 and 5 times a week, 5-minute coaching text message interactions between 3 and 5 times a week, and daily automated text medication reminders requesting a text back to confirm receipt | Youth reported feeling personally connected to their coach via phone calls. Youth who received phone calls appeared to develop deeper relationships with their coach than those who communicated with coaches via text. | Youth in the phone-based group felt motivated to avoid disappointing their coach. Phone calls also helped to disrupt youth's daily lives and served as reminders to engage in the intervention. By contrast, those in the automated support group described how not needing to respond to these reminders meant that they did not need to engage as much. | Relationships formed with coaches via phone call were more personal and likely trusting. It was emotionally difficult for these youth after coaching ended, indicating that they had developed strong relationships. |
| Stiles-Shields et al. (2019) | Weekly 5-min. coaching calls (email if participant could not be reached) with purpose of promoting engagement with the intervention. | Not mentioned | Coaches asked questions like "How could you use this app this week to help your mood?" | Coaches asked questions like "What would be a good goal for using the app this week?" (reciprocity). |
| van Middelaar et al. (2018) | Coaching | Participant "connection" with coach sustained engagement with the platform. | One participant stated that their coach "made" them want to accomplish their goals. Otherwise, accountability modes are not discussed. | To establish trust, participants had an in-person baseline consultation with their assigned coach. Participant "connection" with coach sustained engagement with the platform. A positive and personal tone from coaches boosted participants' motivation to participate (i.e., benevolence). |
| Whiteside et al. (2019) | Weekly sessions with therapist who provides psychoeducation and discusses with family how to apply the psychoeducational material to their child's symptoms. Therapist guides family on facilitating exposures for child. Exposures by therapist or family are recorded in the "check-up" module of the platform. Therapist is able to monitor the check-up page in the web-based portal and provide direction in-session and remotely throughout the week. | Not mentioned | Therapists could review participant activities and exposures logged. One participant stated that the app allowed their child to take responsibility for her own symptom management. | Intervention was designed to be delivered by therapists with expertise in child mental health but not necessarily knowledge of exposure therapy. They received phone training (60-90 min.) and a written manual. |
